# Noninvasive Assessment of Arterial Wall and Soluble ST2 in Patients with Type 2 Diabetes and Coronary Artery Disease

**DOI:** 10.3390/ijms26157561

**Published:** 2025-08-05

**Authors:** Edyta Radzik, Marcin Schulz, Brygida Przywara-Chowaniec, Andrzej Tomasik

**Affiliations:** II Department of Cardiology in Zabrze, Medical University of Silesia, 10 M. Curie-Skłodowska Street, 41-800 Zabrze, Poland; mail.schulz.od44@gmail.com (M.S.); atomasik@sum.edu.pl (A.T.)

**Keywords:** arterial stiffness, diabetes mellitus, vascular age, vascular wall fibrosis, inflammation, sST2, cardiovascular risk, endothelial dysfunction

## Abstract

Diabetes-related pathophysiological processes contribute to endothelial dysfunction, arterial stiffening (AS), hypertension, vascular remodeling, and impaired myocardial perfusion. This study aimed to assess the relationship between arterial wall parameters and sST2 concentration as potential risk factors in type 2 diabetes (T2DM) and investigate sex-related differences. To achieve this, we enrolled 100 patients with suspected or exacerbated coronary artery disease (CAD) and divided them into a T2DM group (n = 58) and a control group (n = 42). Endothelial reactivity (lnRHI), ABI, sST2 levels, and carotid–femoral (cfPWV) and carotid–radial pulse wave velocity (crPWV) were assessed. Coronary angiography was performed in every patient, and epicardial flow and myocardial perfusion were evaluated using QuBE and FLASH. Our results showed that the coronary angiographic findings were similar in both groups. However, T2DM patients had a significantly higher central AS (cfPWV 10.8 ± 2 vs. 9.9 ± 2.7 m/s, *p* < 0.05) and vascular age (70.0 ± 12.3 vs. 61.3 ± 15.4 years, *p* < 0.05), while peripheral AS, RHI, and ABI showed no differences. CfPWV correlated with renal function; higher HbA1c and sST2 levels were additionally associated with advanced vascular age. Notably, central AS and vascular age were higher in men with T2DM but not in women. These findings indicate that T2DM patients exhibit increased central AS and vascular aging, influenced by sST2 levels, suggesting fibrosis as a target for precision medicine in T2DM.

## 1. Introduction

Patients with diabetes are nearly five times more likely to develop heart disease or stroke compared to the general population, with cardiovascular disease being the leading cause of death in this group [1]. Chronic hyperglycemia triggers a cascade of vascular changes, beginning with endothelial dysfunction—the initial stage of both micro- and macrovascular complications. This dysfunction increases vascular permeability and contractility while also stimulating extracellular matrix production [2,3]. Moreover, prothrombotic processes are initiated by upregulating adhesion molecules, further accelerating atherogenesis [1,2,3,4]. Chronic hyperglycemia also contributes to the formation of advanced glycation end products (AGEs), which exacerbate oxidative stress and inflammation, intensify endothelial barrier permeability, promote atherogenesis, and stimulate the proliferation of vascular smooth muscle cells (VSMCs) [5]. Additionally, AGEs interact with the basement membrane, forming cross-links between collagen and proteoglycans, ultimately leading to reduced vascular elasticity and the development of arterial stiffness (AS) [6]. Central AS is a progressive process associated with aging, driven by structural and functional changes in the vascular wall. As large arteries become stiffer, vascular resistance increases, redirecting pulsatile blood flow toward the proximal small arterioles. This exposes them to shear forces, leading to endothelial dysfunction and microcirculatory inflammation [7]. Importantly, a decreased elasticity of the large arteries results in increased afterload and decreased coronary perfusion during diastole, leading to heart failure [8]. AS is further accelerated by pathological conditions such as diabetes, as well as abdominal obesity, hypertension, and dyslipidemia [9]. The impact of comorbidities, genetic predisposition, and lifestyle factors contributes to early vascular aging, leading to an increased cardiovascular mortality [10,11]. In this study, we aimed to investigate several noninvasive parameters of arterial wall structure, function, and integrity in patients with coronary artery disease (CAD) and T2DM.

## 2. Results

### 2.1. Clinical Characteristics of Patients

The clinical characteristics of the study population are presented in Table 1. The study group comprised 58 patients diagnosed with T2DM, of whom 52% were women and 48% were men. Among these patients, 52 (89.7%) were diagnosed with chronic coronary syndrome, 12 (20.7%) with chronic kidney disease (CKD), 54 (93.1%) with arterial hypertension, 50 (86.2%) with hyperlipidemia, and 4 (6.9%) had a history of cerebrovascular stroke. There were no significant differences between the study and control groups regarding sex distribution or age. However, patients with T2DM had a significantly higher body mass index (BMI) compared to those without T2DM. The proportion of former smokers was higher in the control group, while the number of active smokers did not differ significantly between groups. No statistically significant differences were observed between groups in the prevalence of hyperlipidemia or hypertension or the history of ischemic stroke. By contrast, CKD was significantly more prevalent among patients with T2DM and was not observed in any participant without T2DM. The classification of CKD did not distinguish between diabetic kidney disease and the renal impairment of other etiologies.

### 2.2. Assessment of Epicardial Coronary Flow and Myocardial Perfusion

Corrected frame count (cTFC) and FLASH analysis determined the flow through the epicardial coronary arteries. Myocardial perfusion was assessed by QuBE. Data are presented in Table 2 below.

All patients demonstrated comparable angiographic findings of the coronary arteries. Single-vessel CAD was diagnosed in 18 patients (41.1%) without T2DM and 23 patients (39.7%) with T2DM, while two-vessel disease was identified in 14 patients (33.3%) without T2DM and 16 patients (27.6%) with T2DM. In the remaining cases, stenotic lesions were present in all three major coronary arteries. The number of stenotic lesions did not differ significantly between the groups.

Similarly, no statistically significant differences were observed in epicardial flow velocity, assessed by determining the number of frames required for the contrast medium to traverse the vessel’s length (“contrast front”), or in overall coronary flow volume. Myocardial perfusion was comparable in both groups. In contrast to our previous studies, perfusion analysis using QuBE in this study yielded substantially lower values. This discrepancy is likely attributable to the implementation of a different digital data archiving system between the earlier investigations and the present study.

### 2.3. Results of Measurements of Vascular Wall Condition Markers

**Figure 1:**
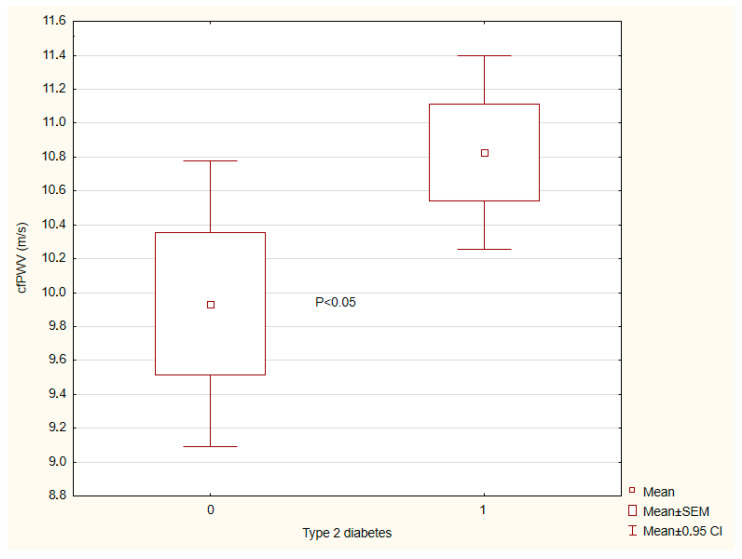
Carotid–femoral pulse wave velocity (cfPWV) depending on presence (1)/absence (0) of type 2 diabetes. There are 42 and 52 in groups (0) and (1), respectively.

**Figure 2:**
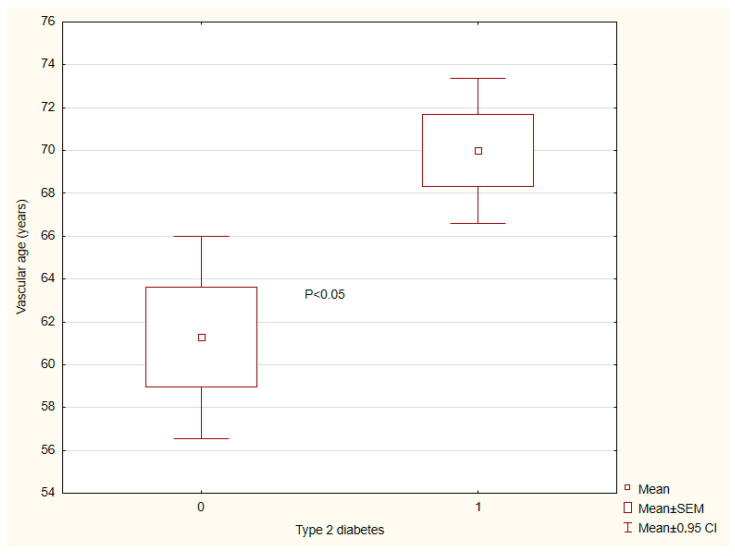
Vascular age depending on presence (1)/absence (0) of type 2 diabetes. There are 42 and 52 patients in groups (0) and (1), respectively.

**Figure 3:**
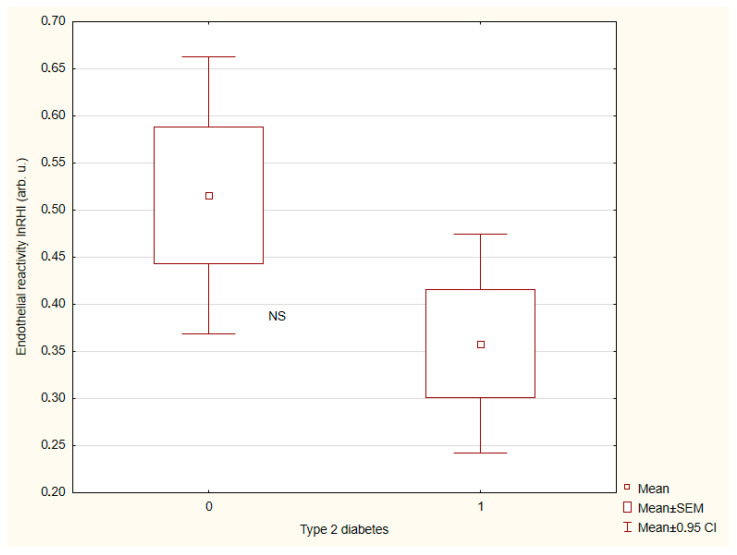
Endothelial reactivity depending on presence (1)/absence (0) of type 2 diabetes. There are 40 and 48 patients in groups (0) and (1), respectively.

**Figure 4:**
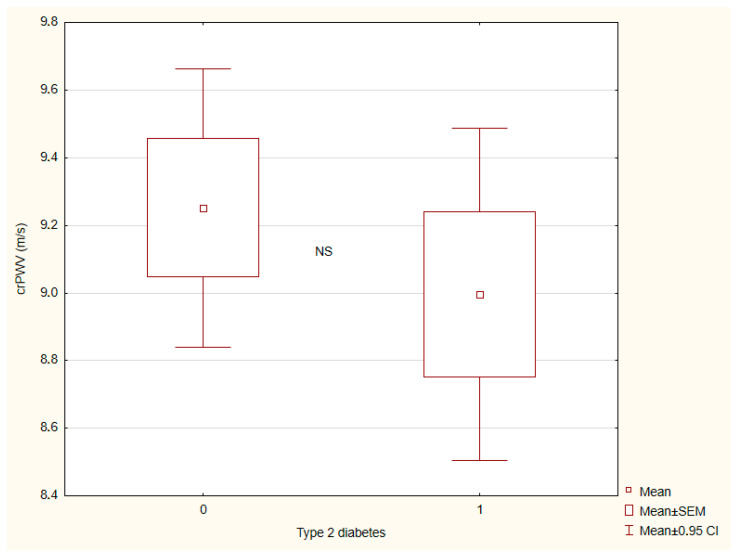
Carotid–radial pulse wave velocity (crPWV) depending on presence (1)/absence (0) of type 2 diabetes. There are 42 and 52 patients in groups (0) and (1), respectively.

The results of vascular wall parameters are presented in Table 3. Patients with tT2DM exhibited a significantly greater central AS than individuals without T2DM (Figure 1) and a more advanced vascular age (Figure 2). By contrast, endothelial reactivity, crPWV, ABI, and blood pressure showed no significant differences between the groups (Figure 3 and Figure 4).

In two patients, the result of LnRHI could not be obtained despite repeated measurement attempts. Due to the complete lack of generation of a spindle-shaped curve after deflation of the cuff on the examined arm and the very irregular curve recording throughout the study period, the program reported that it could not calculate the result. Both patients, nos. 41 and 48, suffered from T2DM; achieved high cfPWV values, 12.7 and 17.0 m/s; and had an improperly controlled CBP of 164/84 and 165/71 mmHg, respectively. Their vascular age was also higher than their biological age by 10 and 12 years, and their sST2 concentrations were 42.13 and 39.38, respectively. Patient no. 41 had a negative history of smoking, while his BMI was 36.2. By contrast, patient no. 48 had a positive history of smoking (60 pack-years), and his BMI was 19.9. Both patients suffered from hyperlipidemia, patient no. 41 had a history of AMI, while patient no. 48 suffered from CKD.

### 2.4. Results of Biochemical Parameter Evaluation

The results from the evaluation of biochemical parameters are presented in Table 4. We showed that patients with diagnosed CAD and T2DM, as expected, have higher levels of glycated hemoglobin and worse renal function. Additionally, we found a higher level of sST2 in patients with diabetes, which may indicate more advanced fibrosis processes in this group of patients.

### 2.5. The Relationship Between Biochemical Parameters and Markers Assessing the Condition of the Vascular Wall

The results of multiple regression for cfPWV and vascular age (dependent variables) with biochemical parameters as independent variables are presented in Table 5. cfPWV depends on the kidneys’ excretory function, while higher HbA1c concentrations and higher sST2 protein concentrations additionally determine more advanced vascular age. The latter two parameters are related to the glycosylation of vascular wall proteins, contributing to changes in its elasticity and more intense fibrosis processes, thus increasing AS.

### 2.6. The Influence of Sex on the Obtained Results

The results of vascular markers and biochemical assessment depending on sex are presented in Table 6, while Table 7 also takes into account the incidence of T2DM. Women and men differed in terms of blood pressure values, especially in the subgroup of women without diabetes, where the highest mean values of systolic blood pressure were observed. Men had significantly higher creatinine levels, which seems to reflect the greater muscle mass in men. The subgroup of men with diabetes had the highest values of cfPWV and vascular age. The values of ABI, endothelial reactivity, and peripheral AS did not differ significantly between the groups.

## 3. Discussion

Previous studies have highlighted the prognostic value of cfPWV in predicting cardiovascular events and its correlation with the extent of atherosclerosis in the coronary arteries [12,13,14]. The incorporation of cfPWV into conventional cardiovascular risk assessment models was associated with reclassification into higher risk categories, particularly among individuals initially categorized as being at low risk [15,16]. The present study focused on patients with established CAD, representing a population with very high cardiovascular risk. In our study, diabetic patients exhibited more advanced central AS and increased vascular age than the control group. The phenomenon of early vascular aging (EVA) in diabetes is multifactorial, driven by impaired endothelial function, microcirculatory disorders, chronic inflammation, insulin resistance, and vascular calcification, often exacerbated by nephropathy and secondary hyperparathyroidism [17]. Consequently, the development of T2DM has been associated with an eight-fold decrease in the likelihood of healthy vascular aging (HVA) [10]. The ARIC (Atherosclerosis Risk in Communities) study demonstrated a strong link between T2DM and increased AS, with the effect comparable to over six years of aging in nonsmokers without T2DM [18].

Our study demonstrated that AS is associated not only with the development of T2DM but also with glycemic control, as measured by HbA1c levels. It is important to note that impaired glucose metabolism and prolonged exposure to hyperglycemia promote collagen cross-linking through non-enzymatic glycation, leading to the development of AS even before the clinical diagnosis of T2DM [19]. Furthermore, the accumulation of AGEs during hyperglycemia increases the activation of the receptor for AGEs (RAGE), which subsequently triggers the release of various profibrotic factors, including transforming growth factor-beta (TGF-β) and connective tissue growth factor (CTGF), while also stimulating the production of extracellular matrix proteins [20]. The Whitehall II study provided further evidence of this relationship, demonstrating that in non-diabetic individuals, a 0.07% increase in HbA1c was associated with a 0.11 m/s increase in cfPWV over five years, accounting for approximately 14% of the mean 5-year cfPWV progression [21]. This suggests that even within normoglycemic ranges, factors related to glucose metabolism can influence the progression of AS. This relationship is clinically significant, as glycemic control represents a modifiable risk factor. Evidence suggests that achieving optimal glycemic control can reduce or slow the progression of AS [22].

Our findings highlight a bidirectional relationship between AS and renal function. Although mean eGFR values significantly differed between groups, both remained within stage G2 (eGFR 60–89 mL/min/1.73 m^2^), indicating that even mild renal dysfunction may influence vascular structure and function. AS was associated with mechanisms such as AGE accumulation, vascular calcification, and activation of the renin–angiotensin–aldosterone system [23], while hyperphosphatemia further contributed to vascular injury through inflammatory and oxidative pathways [24,25]. Conversely, increased AS may impair renal function by elevating pulse pressure and transmitting hemodynamic stress to the renal microcirculation [26]. The observation of higher PWV in the central arteries, particularly in patients with CKD and T2DM, supports this association, whereas peripheral AS (crPWV) showed no significant correlation with renal function.

Elevated HbA1c levels and declining renal function in patients with T2DM are associated with extracellular matrix remodeling and the intensification of pathological fibrosis within the vascular wall, which may explain the significant differences in sST2 levels observed between groups. We demonstrated that sST2 levels are significantly higher in patients with both T2DM and CAD compared to those without T2DM and correlate with the severity of central AS, expressed as vascular age. Interleukin-33 (IL-33), the ligand for ST2, plays a key role in modulating inflammatory and fibrotic responses in the myocardium. Under pathological conditions such as ischemia or pressure overload, cardiac fibroblasts release proinflammatory cytokines that contribute to myocardial fibrosis and dysfunction. IL-33, also produced by fibroblasts, exerts cardioprotective effects by binding to the transmembrane ST2L receptor, leading to the activation of MAPK and NF-κB signaling pathways, which attenuate hypertrophy, mitochondrial dysfunction, and apoptosis in cardiomyocytes. By contrast, the sST2 functions as a decoy receptor that neutralizes IL-33, thereby inhibiting its interaction with ST2L and abolishing its protective effects [27]. In previous studies, elevated sST2 levels have been linked to central arterial stiffness, as evidenced by their association with increased aortic pulse pressure measured before coronary angiography [28]. This may result from fibrotic remodeling of the vascular wall, supported by in vitro findings showing that sST2 stimulates vascular smooth muscle cells to produce type I collagen, fibronectin, and profibrotic mediators [29]. Moreover, in a study by Miller et al. investigating the impact of this pathway on vascular wall integrity, it was reported that in a murine model, the administration of sST2 resulted in significantly larger atherosclerotic plaques in the Valsalva sinuses compared to controls, whereas IL-33 infusion led to a reduction in plaque size [30,31,32]. In contrast to central AS, peripheral AS assessed by crPWV showed no association with the presence of T2DM. Previous studies have reported inconsistent findings regarding the relationship between peripheral AS and CAD [33,34,35], and the link between T2DM and peripheral AS remains uncertain. The ARIC study found reduced peripheral AS in T2DM patients, suggesting distinct vascular remodeling mechanisms in large elastic versus peripheral arteries [18]. Conversely, a meta-analysis by Liang et al. observed higher crPWV values in T2DM patients compared to healthy individuals, though the difference was not statistically significant [36]. These discrepancies suggest that pathological processes may affect PWV differently depending on the vascular region [37,38,39,40,41,42]. In our study, women exhibited a higher cfPWV and vascular age compared to men; however, these differences did not reach statistical significance. Women also had significantly poorer blood pressure control. In both men and women with T2DM, cfPWV and vascular age were elevated compared to men without T2DM. Among women, no significant differences in AS were observed between those with and without T2DM, suggesting that within the studied age group, T2DM may not substantially influence AS progression in this population. However, the number of women without T2DM in our cohort was relatively small, limiting the statistical power of this comparison. Therefore, our findings indicate a trend but do not allow for definitive conclusions.

By contrast, in men, the presence of T2DM was significantly associated with elevated cfPWV and vascular age, indicating that T2DM is a major determinant of AS in this group. Previous studies have reported more advanced AS in women than in men of similar age and blood pressure profiles [41]. However, Zhang et al. demonstrated that while arterial stiffness is higher in women over the age of 60 with normal glucose levels, the presence of prediabetes or T2DM attenuates this sex-related difference, resulting in comparable AS severity between men and women [42].

Our study found that lnRHI was lower in patients with T2DM compared to controls, though the difference was not statistically significant. In addition, no significant differences were found between groups in terms of ABI or lnRHI. While this may suggest the absence of peripheral perfusion deficits or endothelial dysfunction in patients with T2DM, the limited statistical power must be considered. Both lnRHI and ABI are characterized by considerable variability and may exhibit relatively low sensitivity in detecting early vascular changes. Therefore, the lack of statistical significance should be interpreted with caution.

Previous research indicates that RHI is reduced in individuals with cardiovascular diseases [43]; however, its role as a risk factor for MACE and its significance in T2DM remain uncertain [44,45]. Studies have linked reduced RHI to advanced multivessel CAD, impaired coronary reserve post revascularization, and diminished myocardial perfusion in the general population [43,46], but no such correlation has been observed in T2DM patients [47]. Research on endothelial dysfunction in T2DM has yielded conflicting results. While some studies report significantly lower RHI in T2DM, suggesting that endothelial dysfunction emerges with overt T2DM [48], others indicate that obesity, rather than T2DM, is the primary determinant of RHI reduction [49]. These findings suggest that RHI measurement may not be sufficiently sensitive to detect endothelial dysfunction in T2DM, and its prognostic value in this population remains uncertain. Future studies with larger cohorts and sample size calculations tailored to detect more subtle effects are warranted.

Although ABI has limitations in detecting PAD among patients with T2DM, both abnormally low and high ABI values have been associated with increased cardiovascular risk and mortality [50]. Previous studies indicate that ABI values above 1.4 are suggestive of AS [48], but in patients with T2DM, these measurements may be falsely elevated despite the presence of PAD [51]. Clairotte et al. recommend redefining the normal ABI range for individuals with T2DM, suggesting a PAD diagnosis at values below 1.0–1.1, due to the influence of medial arterial calcification and increased AS in this group [52]. Our study found no significant difference in ABI between the study and control groups; however, AS was significantly more prevalent in the study group, suggesting that ABI may overestimate lower limb arterial status in certain populations. As described above, the lack of statistically significant differences may result from the limited power of the study and the relatively small sample size.

Our study highlights sST2 as a valuable biomarker for assessing AS and vascular aging in patients with T2DM. Elevated sST2 levels indicate underlying vascular fibrosis, which plays a crucial role in the progression of diabetic vascular damage. Standardized and rapid sST2 assays can be integrated into routine clinical practice to identify T2DM patients at higher risk of accelerated vascular aging. Therapeutic strategies targeting vascular fibrosis—such as pioglitazone [53], GLP-1 receptor agonists like liraglutide [54], and agents modifying extracellular matrix remodeling [55] or epigenetic regulation [56]—may hold promise in improving vascular outcomes in diabetic patients. Monitoring sST2 levels could help evaluate the response to such interventions and personalize treatment. These findings support the integration of sST2 into clinical practice as part of a precision medicine approach to vascular health in diabetes.

## 4. Materials and Methods

This single-center prospective study enrolled 100 patients aged 50 to 80 years who were hospitalized between 1 October 2019, and 31 October 2022, at the 2nd Department and Clinical Department of Cardiology in Zabrze (Katowice, Silesia, Poland) for invasive diagnostics due to suspected or worsening CAD. Patients who underwent coronary angiography were invited to participate in this study. The study group consisted of patients diagnosed with T2DM, while the control group comprised healthy individuals matched for sex and age (±2 years). In addition to the standard consent related to healthcare provision, all participants received detailed written information regarding the study and provided informed written consent for participation.

The exclusion criteria: Inability to independently provide consent to participate in the study, history of surgical or percutaneous procedures involving the aorta and peripheral arteries, presence of arteriovenous fistulas, presence of aneurysms and pseudoaneurysms, presence of ulcers or necrotic changes in the lower limbs, history of limb amputation, active neoplastic process, active inflammatory state (clinically or biochemically), and lower limb edema.

### 4.1. Clinical Evaluation

A detailed medical interview was conducted with each patient included in this study regarding current ailments, concomitant diseases, previous surgeries, stimulants, and pharmacotherapy. We measured body weight and height and calculated BMI with the Seca 287 stadiometer (Seca GmbH, Hamburg, Germany).

### 4.2. Analyzed Parameters

#### 4.2.1. sST2

Venous blood was collected from each patient in the morning into a tube with EDTA. Patients were fasting at the time of collection. The concentration of sST2 was assessed using the Aspect Reader device (Critical Diagnostics, San Diego, CA, USA).

#### 4.2.2. Biochemical Tests

In this study, we analyzed the following laboratory tests, which were retrieved from patients’ electronic records: peripheral blood morphology, creatinine, eGFR, high-sensitivity troponin T (hsTnT), NT-pro-BNP, and HbA1c.

### 4.3. Coronary Angiography and Angiogram Analysis

Coronary angiography was performed predominantly from right radial access. The procedure was recorded digitally using Philips Integris Azurion and Philips Allura systems at a frame rate of 30 frames per second. Low-osmolar contrast agents, Ultravist and Visipaque, were utilized for imaging. In cases where significant coronary artery stenosis was identified, percutaneous coronary angioplasty was performed to restore or improve vessel patency. The angiographic criterion for angioplasty was a luminal narrowing of more than 70%, except for the left main coronary artery (LM), where a threshold of >50% was applied. The severity of CAD was assessed both subjectively and objectively. Qualitative evaluation was based on the number of affected vessels. Additionally, quantitative assessments included coronary artery flow analysis using the FLASH protocol (Fluoroscopy-Assisted Scoring of Myocardial Hypoperfusion), myocardial perfusion assessment with QuBE software (version 20091116_v1, SourceForge, released 15 November 2009), and epicardial flow evaluation via the corrected TIMI frame count (cTFC). All angiographic studies were archived in the hospital’s RIS system. QuBE and FLASH data analysis was conducted retrospectively on archived records using the INFINITT viewer and the QuBE application (https://qube.sourceforge.net). Blood flow velocity in the coronary arteries, calculated using FLASH protocol, is expressed as the volume of blood passing per unit time. The vessel contour was manually delineated for precise measurement [57,58].

### 4.4. Blood Pressure Assessment

This study began after a 10 min rest using the WatchBP Office ABI device (Microlife Corporation, Taipei, Taiwan), with appropriately sized cuffs based on body weight and arm circumference. Three consecutive blood pressure readings were taken simultaneously on both arms; the device then calculated the average value for each arm. The measurement from the arm with the higher average systolic (SBP) and diastolic (DBP) values was used in the study.

### 4.5. ABI Assessment

The ankle–brachial index (ABI) was assessed using the WatchBP Office ABI device with the oscillometric method, validated by the European Society of Hypertension and the American Heart Association. Blood pressure cuffs were placed on the left arm and lower limb, and automated ABI measurements were taken sequentially for standardized data acquisition.

### 4.6. Arterial Stiffness Assessment

The measurement was conducted in the morning in an isolated room (22 °C, dimmed lighting). Participants fasted for 8 h, avoiding smoking, caffeine, and medications. The Complior (Alam Medical, Saint-Quentin-Fallavier, France) device and software (Complior, version v1.9.5.5) were used, with participants in a supine position. Complior generated key hemodynamic parameters, including carotid–femoral pulse wave velocity (cfPWV), carotid–radial pulse wave velocity (crPWV), augmentation index (AIx), central blood pressure, vascular age, and pulse wave analysis (PWA). Vascular age was calculated based on cfPWV, following an international study of individuals without overt cardiovascular disease (CVD), type 2 diabetes mellitus (T2DM), or antihypertensive/lipid-lowering therapy. Reference cfPWV values were established for optimal and normal blood pressure groups, adjusted for age, and for hypertension subgroups, excluding CVD cases [59].

### 4.7. Endothelial Function

The assessment of endothelial reactivity was conducted under the same conditions as arterial stiffness evaluation. Patients trimmed their fingernails (≤5 mm) and removed jewelry. Measurements were performed using the EndoPAT2000 device (Itamar Medical Ltd., Caesarea, Israel). and software (EndoPAT2000, version 3.7.2).A sphygmomanometer cuff was placed 4–5 cm above the antecubital fossa, while plethysmographic sensors were positioned on both index fingertips. The device recorded baseline pulsatile blood volume changes for 5 min. Blood flow was then restricted by inflating the cuff to ≥60 mmHg above systolic blood pressure (SBP) or 200 mmHg, whichever was higher. After 5 min, deflation restored flow, stimulating endothelial nitric oxide synthase (eNOS) and vasodilation, visualized as a spindle-shaped waveform. Microcirculation recovery was monitored for 10 min. The device automatically calculated the reactive hyperemia index (RHI) and its logarithmic form (lnRHI), both indicators of endothelial function [60].

### 4.8. Statistical Analysis

Statistical analysis of the data was performed using both parametric and non-parametric comparative tests, while the normality of continuous variable distributions was assessed using the Kolmogorov–Smirnov test and the Chi-square test [61,62,63]. In the case of a normal distribution, the statistical significance of differences between independent continuous variables was evaluated using ANOVA and Student’s *t*-test. For dependent data, comparisons were made using the Kruskal–Wallis analysis of variance, and if significance was detected, the paired-samples Student’s *t*-test was performed. In multiple analyses of the same variables, Bonferroni correction was applied. Multiple regression analysis was also used to determine factors describing AS in the studied population.

For non-normally distributed continuous variables, statistical analysis was performed using the Wilcoxon signed-rank test or the Mann–Whitney U test. Multiple groups were compared using the Kruskal–Wallis analysis of variance, and if significance was detected, multiple comparisons of mean ranks were conducted. Qualitative data were analyzed using the Chi-square test with Yates’ correction. Additionally, multiple regression analysis was used to identify the independent variables best describing the assessed dependent variable. The results are presented as frequencies (for qualitative variables) or as mean ± standard deviation for quantitative variables. HbA1c data in non-diabetic, control group were incomplete, so we eventually, have completed them according to the procedure described in “Statistical Methods” [63] and “Principled missing data methods for researchers” [64].

## 5. Conclusions

Elevated central AS in the population of patients with T2DM may be interpreted as an additional cardiovascular risk factor. The correlation between HbA1c levels, renal function, and sST2 concentrations with AS highlights the significant role of fibrotic processes in developing arterial stiffening. This suggests that targeting fibrosis attenuation could serve as a therapeutic strategy in diabetic patients suffering from CAD. The evaluated parameters of peripheral circulation (RHI, ABI, and crPWV) did not differ significantly between the study and control groups, indicating that they are unsuitable cardiovascular risk markers in the population of patients with T2DM and CAD. The presence of T2DM has a greater impact on the development of AS in men than in women. Therefore, reducing arterial stiffness represents a particularly important therapeutic goal in this subgroup of patients.

## 6. Study Limitations

This study was conducted at a single center and included only Caucasian participants, which limits the generalizability of the findings to other populations with different ethnic backgrounds.

The study population consisted of individuals referred for invasive diagnostics due to suspected coronary artery disease, representing a higher-risk group compared to the general population. Due to the low number of participants without confirmed coronary disease, the results cannot be extrapolated to the general population.

The overall sample size was relatively small, particularly in the context of secondary outcomes such as lnRHI and ABI. Both parameters are characterized by high variability and subtle differences between groups, which may have reduced the statistical power to detect significant associations. Therefore, the lack of statistical significance for these endpoints should be interpreted with caution.

Some subgroup analyses, such as those involving women without type 2 diabetes, included very few participants. These findings are therefore considered exploratory and do not support definitive conclusions.

Changes in angiographic data archiving and the resulting digital image compression limited the ability to interpret myocardial perfusion when assessed using the QuBE software and hindered its correlation with peripheral vascular parameters.

The cross-sectional nature of the study precludes the assessment of causal relationships. Prospective follow-up would be more appropriate for establishing cause–effect associations.

## Figures and Tables

**Table 1:** Clinical characteristics of patients.

	T2DM (−) n = 42	T2DM (+) n = 58	Significance
Sex, women/men	28/14	28/30	NS
Age, years (X ± SD)	66.5 ± 7.0	69.4 ± 7.5	NS
Height, m (X ± SD)	1.69 ± 0.08	1.64 ± 0.09	*p* < 0.05
Body mass, kg (X ± SD)	80.8 ± 12.6	86.9 ± 16.6	*p* < 0.05
BMI ^1^, kg/m^2^ (X ± SD)	28.4 ± 4.7	33.0 ± 6.3	*p* < 0.001
Active smokers, n (%)	10 (23.8)	12 (20.7)	NS
Former smokers, n (%)	24 (57.1)	20 (34.5)	*p* < 0.05
Family health history, n (%)	14 (33.3)	32 (55.2)	NS
Concomitant diseases			
Chronic coronary syndrome, n (%)	38 (90.5)	52 (89.7)	NS
Hyperlipidemia, n (%)	30 (71.4)	50 (86.2)	NS
Arterial hypertension, n (%)	34 (80.9)	54 (93.1)	NS
Ischemic stroke, n (%)	4 (9.5)	4 (6.9)	NS
CKD ^2^, n (%)	0 (0)	12 (20.7)	*p* < 0.01
Drugs			
ACEI ^3^, n (%)	36 (85.0)	40 (69.0)	NS
ARB ^4^, n (%)	6 (14.3)	14 (24.1)	NS
CCB ^5^, n (%)	12 (28.4)	32 (55.2)	*p* < 0.05
Beta-blocker, n (%)	38 (90.5)	52 (89.7)	NS
Diuretic, n (%)	14 (33.3)	32 (60.3)	NS
Potassium-sparing diuretic, n (%)	8 (19.0)	12 (20.7)	NS
ASA ^6^, n (%)	36 (85.0)	56 (95.6)	NS
Clopidogrel, n (%)	22 (52.4)	32 (60.3)	NS
Ticagrelor, n (%)	4 (9.5)	6 (10.3)	NS
Fibrate, n (%)	0 (0)	12 (20.7)	*p* < 0.01
Statin, n (%)	40 (95.2)	50 (86.2)	NS
OAC ^7^, n (%)	2 (4.8)	2 (3.4)	NS
Metformin, n (%)	0	42	
Sulfonyl ^8^, n (%)	0	18	
SGLT2 inhibitor, n (%)	2	10	
Insulin, n (%)	0	24	

^1^ Body mass index. ^2^ Chronic kidney disease. ^3^ Angiotensin-converting enzyme inhibitor. ^4^ Angiotensin receptor blocker. ^5^ Calcium channel blocker. ^6^ Acetylsalicylic acid. ^7^ Oral anticoagulants. ^8^ Sulfonylurea derivative.

**Table 2:** Results of measurements of coronary circulation parameters.

	T2DM (−) n = 42	T2DM (+) n = 58	Significance
Number of narrowed coronary arteries, n (%)			
1	18 (42.9)	23 (39.7)	NS
2	14 (33.3)	16 (27.6)	
3	10 (23.8)	19 (32.7)	
LAD ^1^			
cTFC, X ± SD	26.3 ± 8.4	24.6 ± 9.3	NS
FLASH, mL/min, X ± SD	48.4 ± 15.7	50.3 ± 19.8	NS
QuBE, arb. units X ± SD	3.5 ± 2.3	3.8 ± 3.6	NS
Cx ^2^			
cTFC, X ± SD	18.4 ± 6.5	19.8 ± 7.5	NS
FLASH, mL/min, X ± SD	42.5 ± 14.9	40.4 ± 16.8	NS
QuBE, arb. units X ± SD	3.1 ± 4.3	2.9 ± 2.6	NS
RCA ^3^			
cTFC, X ± SD	20.1 ± 7.5	18.8 ± 8.7	NS
FLASH, mL/min, X ± SD	44.7 ± 18.2	47.1 ± 18.4	NS
QuBE, arb. units X ± SD	4.2 ± 3.5	4.9 ± 3.1	NS

^1^ Left anterior descending artery. ^2^ Circumflex artery. ^3^ Right coronary artery.

**Table 3:** Vascular wall parameters in patients with and without T2DM.

	T2DM (−) n = 42	T2DM (+) n = 58	Significance
SBP ^1^, mmHg, X ± SD	138.6 ± 19.4	143.2 ± 19.0	NS
DBP ^2^, mmHg, X ± SD	80.0 ± 10.4	80.9 ± 10.3	NS
ABI ^3^ right leg, X ± SD	1.23 ± 0.11	1.22 ± 0.18	NS
ABI ^3^ left leg, X ± SD	1.25 ± 0.12	1.22 ± 0.21	NS
LnRHI ^4^, X ± SD	0.52 ± 0.47	0.36 ± 0.4	NS
cfPWV ^5^, m/s, X ± SD	9.9 ± 2.7	10.8 ± 2.1	<0.05
CrPWV ^6^, m/s, X ± SD	9.3 ± 2.7	9.0 ± 2.1	NS
Vascular age, years, X ± SD	61.3 ± 15.4	70.0 ± 12.3	<0.05

^1^ Systolic blood pressure. ^2^ Diastolic blood pressure. ^3^ Ankle–brachial index. ^4^ Natural logarithm of the reactive hyperemia index. ^5^ Carotid–femoral pulse wave velocity. ^6^ Carotid–radial pulse wave velocity.

**Table 4:** Results of biochemical parameter evaluation.

	T2DM (−) n = 42	T2DM (+) n = 58	Significance
HbA1C, %, X ± SD	5.8 ± 0.1	8.2 ± 1.3	*p* < 0.001
Creatinine, µmol/L, X ± SD	73.2 ± 15.0	88.9 ± 36.2	*p* < 0.05
eGFR, mL/min, X ± SD	82.1 ± 7.7	69.0 ± 21.0	*p* < 0.001
sST2, ng/mL, X ± SD	22.2 ± 6.6	34.7 ± 27.7	*p* < 0.01

**Table 5:** The relationship between biochemical parameters and markers assessing the condition of the vascular wall.

Dependent Variable	Independent Variable	β	t	P
cfPWV	HbA1C	0.18	1.68	NS
	Creatinine	1.12	2.65	*p* < 0.01
	eGFR	−0.94	−2.14	*p* < 0.01
	sST2	0.11	1.09	NS
	Regression Summary			
	Multiple R = 0.47			
	Multiple R^2^ = 0.16			
	Adjusted R^2^ = 0.09			
	*p* < 0.05			
Vascular age	HbA1C	0.15	1.35	NS
	Creatinine	0.45	1.9	*p* < 0.05
	eGFR	−0.53	−2.25	*p* < 0.05
	sST2	0.53	2.32	*p* < 0.05
	Regression Summary			
	Multiple R = 0.36			
	Multiple R^2^ = 0.13			
	Adjusted R^2^ = 0.09			
	*p* < 0.01			

**Table 6:** Comparison of vascular wall markers and biochemical parameters according to sex.

	Men n = 56	Women n = 44	Significance
SBP, mmHg, X ± SD	137.4 ± 16.4	146.2 ± 21.2	*p* < 0.01
DBP, mmHg, X ± SD	79.6 ± 8.3	81.7 ± 12.3	NS
ABI right leg, X ± SD	1.22 ± 0.15	1.23 ± 0.16	NS
ABI left leg, X ± SD	1.23 ± 0.17	1.24 ± 0.19	NS
Endothelial reactivity, lnRHI, X ± SD	0.44 ± 0.39	0.42 ± 0.49	NS
cfPWV, m/s, X ± SD	10.2 ± 2.6	10.7 ± 2.1	NS
CrPWV, m/s, X ± SD	9.1 ± 1.7	9.1 ± 1.5	NS
Vascular age, years, X ± SD	63.9 ± 15.9	69.1 ± 10.9	NS
HbA1C, %, X ± SD	7.0 ± 1.4	7.5 ± 1.7	NS
Creatinine, µmol/L, X ± SD	89.7 ± 31.6	71.8 ± 24.8	*p* < 0.005
eGFR, mL/min X ± SD	75.3 ± 16.7	73.5 ± 19.6	NS
sST2, ng/mL, X ± SD	28.2 ± 10.8	31.1 ± 31.3	NS
SBP, mmHg, X ± SD	137.4 ± 16.4	146.2 ± 21.2	*p* < 0.01

**Table 7:** Comparison of vascular wall assessment markers depending on sex and type 2 diabetes.

	Men		Women		Significance
	T2DM (−) n = 28 (1)	T2DM (+) n = 28 (2)	T2DM (−) n = 14 (3)	T2DM (+) n = 30 (4)	Kruskal-Willis ANOVA
SBP, mmHg, X ± SD	132.3 ± 14.4	142.8 ± 17.0	151.1 ± 21.8	143.5 ± 20.8	*p* < 0.01 (*p* < 0.005 1 vs. 3)
DBP, mmHg, X ± SD	79.0 ± 8.7	80.2 ± 8.0	81.9 ± 13.2	81.6 ± 12.1	NS
ABI right leg, X ± SD	1.24 ± 0.11	1.2 ± 0.18	1.21 ± 0.1	1.24 ± 0.19	NS
ABI left leg, X ± SD	1.29 ± 0.13	1.17 ± 0.17	1.18 ± 0.08	1.27 ± 0.22	NS
Endothelium reactivity, lnRHI, X ± SD	0.47 ± 0.42	0.4 ± 0.35	0.63 ± 0.54	0.33 ± 0.44	NS NS
cfPWV, m/s, X ± SD	9.5 ± 2.9	11.0 ± 2.0	10.8 ± 2.1	10.7 ± 2.1	*p* < 0.01 (*p* < 0.01 1 vs. 2 and *p* < 0.05 1 vs. 4)
crPWV, m/s, X ± SD	9.2 ± 1.4	9.0 ± 1.9	9.4 ± 1.6	9.0 ± 1.6	NS
Vascular age, years, X ± SD	57.1 ± 14.8	71.3 ± 13.8	69.7 ± 12.4	68.7 ± 10.2	*p* < 0.01 (*p* < 0.01 1 vs. 2 and *p* < 0.05 1 vs. 4)

## Data Availability

Data are contained within the article.

## Abbreviations

ABI	Ankle–brachial index
AGEs	Advanced glycation endproducts
ARIC	Atherosclerosis Risk in Communities
AS	Arterial stiffness
baPWV	Brachial ankle pulse wave velocity
CAD	Coronary artery disease
cfPWV	Carotid–femoral pulse wave velocity
crPWV	Carotid–radial pulse wave velocity
cTFC	Corrected TIMI frame count
CVD	Cardiovascular disease
Cx	Circumflex artery
EVA	Early vascular aging
faPWV	Femoral ankle pulse wave velocity
FHS	Framingham Heart Study
FLASH	Fluoroscopy-assisted scoring of myocardial hypoperfusion
Il33	Interleukin 33
LAD	Left anterior descending artery
MBG	Myocardial blush grade
MESA	Multi-Ethnic Study of Atherosclerosis
PAD	Peripheral artery disease
QuBE	Quantitative blush evaluator
RCA	Right coronary artery
RHI	Reactive hyperemia index
RIS	Radiology Information System
sST2	Soluble suppression of tumorgenicity 2
TGF-β	Transforming growth factor
TIMI	Thrombolysis in myocardial infarction
T2DM	Type 2 diabetes mellitus
VSCM	Vascular smooth muscle cell
